# Mitochondrial Dysfunction Plays a Relevant Role in Heart Toxicity Caused by MeHg

**DOI:** 10.3390/toxics12100712

**Published:** 2024-09-30

**Authors:** Marcia Gracindo Silva, Camila Guerra Martinez, Joao Paulo Cavalcanti de Albuquerque, André Luiz Gouvêa, Monica Maria Freire, Leidiane Caroline Lauthartte, Julio Mignaco, Wanderley Rodrigues Bastos, Elisabete Cesar de Mattos, Antonio Galina, Eleonora Kurtenbach

**Affiliations:** 1 Instituto de Biofísica Carlos Chagas Filho, Universidade Federal do Rio de Janeiro, Rio de Janeiro 21941-902, RJ, Brazilalgouvea@bioqmed.ufrj.br (A.L.G.); kurten@biof.ufrj.br (E.K.); 2 Instituto de Bioquímica Médica Leopoldo de Meis, Universidade Federal do Rio de Janeiro, Rio de Janeiro 21941-902, RJ, Brazil; 3 Laboratório de Biogeoquímica Ambiental Wolfgang C. Pfeiffer, Universidade Federal de Rondônia, Porto Velho 76801-974, RO, Brazil; 4 Ecodata Exames Médicos Ltd., Rio de Janeiro 22270-018, RJ, Brazil

**Keywords:** methylmercury, heart mitochondria, electrocardiography, echocardiography, ergospirometry, oxidative phosphorylation system

## Abstract

The effects of methylmercury (MeHg) on exposed populations are a public health problem. In contrast to widely studied neurological damage, few cardiovascular changes have been described. Our group evaluated the cardiotoxicity of a cumulative dose of 70 mg.kg^−1^ fractioned over a 14-day exposure period in mice (MeHg70 group). The effects of MeHg on proteins relevant to cardiac mitochondrial function were also investigated. The results obtained showed a reduction in oxygen consumption in the two settings. In cardiac tissue samples in oxygraphy studies, this reduction was related to a lower efficiency of complexes II and V, which belong to the oxidative phosphorylation system. In vivo, mice in the MeHg70 group presented lower oxygen consumption and running tolerance, as shown by ergometric analyses. Cardiac stress was evident in the MeHg70 group, as indicated by a marked increase in the level of the mRNA encoding atrial natriuretic peptide. Electrocardiogram studies revealed a lower heart rate at rest in the animals from the MeHg70 group, as well as prolonged left ventricular depolarisation and repolarisation. Through echocardiographic analysis, reductions in the left ventricular ejection fraction and left ventricular wall thickness of approximately 10% and 20%, respectively, were detected. These results indicate that the oral intake of MeHg can decrease cardiac function and oxidative metabolism. This finding highlights the importance of monitoring MeHg levels in humans and animals in contaminated areas, as well as periodically carrying out cardiac function tests.

## 1. Introduction

Mercury (Hg) is ubiquitously distributed and is considered by the World Health Organization (WHO) to be one of the top ten chemical concerns for public health [1]; it undergoes natural cycling in the environment, which is frequently modified by anthropic actions such as mining and industrial emissions [2,3]. In certain ecosystems, such as the Amazon tropical rainforest, Hg circulates among the soil, plants and atmosphere; it can be deposited in river sediments, where it undergoes bacterial conversion from an ionic form to a metalorganic form, methylmercury (MeHg) [4]. MeHg is promptly absorbed by organisms such as fish and accumulates in their tissues [5]. For the inhabitants of the Amazon, a large part of their protein intake comes from the consumption of fish, and high levels of MeHg, above those considered safe by the WHO, have been detected in tissue samples from riverside residents [6,7,8].

MeHg bioaccumulation has been detected in samples of fishes consumed by the Amazonia population [9,10]. For example, the weekly consumption of approximately 1 kg of “dourada” (Brachyplatystoma flavicans) contaminated with approximately 3.2 MeHg.kg^−1^ of MeHg by an Indaiatuba resident (Pará state, Brazil) could lead to the ingestion of nearly 166 mg of MeHg/year [10]. Orally ingested MeHg is almost completely absorbed from the gastrointestinal tract and circulates in the bloodstream until it reaches the target organs [11].

In humans, neurological damage associated with MeHg has been well characterised since the episodes of collective intoxication in Minamata and Iraq [12,13].

Subsequently, interest in the effects of mercury compounds in other systems, such as cardiovascular systems, has increased. Park et al. (2013) described the association between high concentrations of total mercury in urine (0.51 µg/L) and hypertension in the United States adult population [14]. In rats, Wildeman et al. (2015) reported that, four weeks after exposure to MeHg in drinking water, MeHg significantly increased blood pressure at doses from 10 to 1100 µg MeHg.kg^−1^ [15].

In addition to hypertension, MeHg has also been associated with coronary artery disease (CAD). In Finland, where fish consumption is high, several studies have shown a correlation between MeHg and CAD. Salonen et al. (1995) described that men with higher hair mercury content (2 µg/g) had a 2.0-fold risk of developing CAD [16]. Virtanen et al. (2005) described that men with higher MeHg hair (>2 µg/g) had a 1.6-fold risk of acute coronary event [17]. Tajik et al. (2019) described that hair mercury concentration was directly associated with the occurrence of exercise-induced myocardial ischaemia in men (62% increase in odds) [18].

The effects of organic mercury compounds on cardiac electrical conduction after human poisoning have also been described. Jalili and Abasi (1961) evaluated in Iraq 26 patients who suffered mercury poisoning (a fungicide used for seed-borne diseases of cereals, ethyl mercury p-toluene sulphonanilide) and described that almost all of them presented electrocardiographic abnormalities as ST depression and T wave changes [19]. Dahhan and Orfally, also in Iraq (1964), evaluated 42 patients who suffered ethyl mercury poisoning and described that almost all of them presented electrocardiographic abnormalities such as ST depression, T wave changes, QT prolongation and arrhythmias [20].

Grandjean et al. (2004), in children and adolescents, described that prenatal MeHg exposure was associated with decreased sympathetic and parasympathetic modulation of the heart rate variability (autonomic unfavourable modulation, which is a well-known risk factor for sudden death) [21,22].

Cardiomyocytes have a high oxygen consumption rate and a substantial density of mitochondria (approximately 30% of the cell volume). The heart consumes a high quantity of oxygen and fatty acids for ATP production [23,24]. Mitochondrial dysfunction is linked aetiologically to many heart diseases [25,26]. There is much interest in understanding the mechanisms involved in heart dysfunction, as well as in developing heart dysfunction models, as the development of drugs able to modulate these responses could be a promising way to ameliorate cardiovascular damage. Heart mitochondria could potentially interact with MeHg, which has an affinity for thiol (-SH) groups present in biomolecules, such as the amino acid cysteine. Interactions between MeHg and mitochondrial proteins, such as those composing respiratory system complexes [27], and antioxidant defence systems, including glutathione peroxidase [28] and superoxide dismutase [29], could be critical targets that signal cardiovascular derangement. Cambier et al. [30] reported that after 49 days of feeding zebrafish MeHg-contaminated food, the activity of complex IV (cytochrome c oxidase) was reduced by 60 ± 18%, and a substantial loss of the coupling capacity between oxygen consumption and ATP production in permeabilised red skeletal fibres was detected. These effects occurred despite no signs of interference in complex V (ATP synthase) assembly or changes in ATP hydrolytic activity. Therefore, other components of the phosphorylation system could be directly involved in reducing the ATP/oxygen ratio. Truong et al. (2015) described a decrease in cellular NADH production (~2-fold change), loss in cell viability (~30%) and an increase in reactive oxygen species production (~5-fold change) in AC16 (human ventricle cell line) and H9C2 (rat cardiac myoblast cell line) after incubation with 10 µM MeHg for 24 h [31]. These findings were dose-dependent and displayed species-specific features. However, the studies could not resolve the gap in the knowledge of the effects of MeHg on the heart. After conducting a literature search, we did not find any reports of cardiac function assessments using a combination of electrocardiography, ergometry and echocardiography. These tests can be used serially in human populations at risk from continued exposure to mercury. The aim of this study was to investigate the effects of MeHg in the hearts of a mouse mitochondrial electron-transfer system (ETS) and an oxidative phosphorylation system (OXPHOS) and to estimate their effects on heart function in vivo.

## 2. Materials and Methods

### 2.1. Study Design

**Figure 1:**
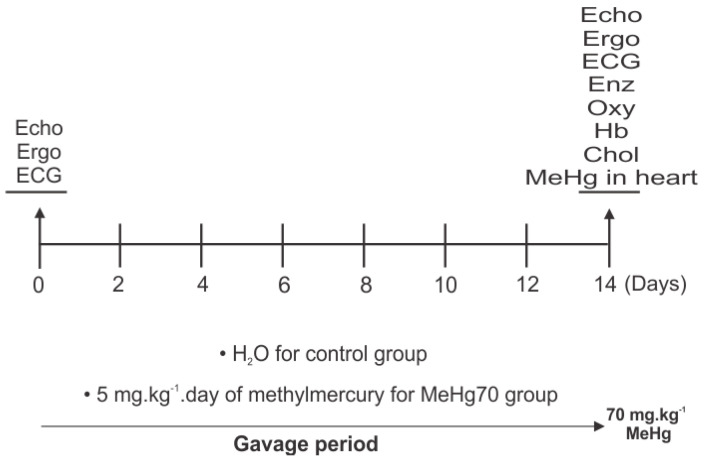
Study design: Heart function pretests, gavage periods and post-tests are indicated; echocardiography (Echo), ergospirometry (Ergo) and electrocardiography (ECG). Fourteen days after MilliQ ultrapure water or MeHg administration, the mice were euthanised under anaesthesia, and the hearts were collected to assess mRNA content via real-time PCR (qPCR) and MeHg concentration analyses. Additionally, in cardiac tissue samples, mitochondrial function was evaluated by high-resolution oxygraphy (Oxy), and enzymatic activity assays (Enz) were performed for the following enzymes: citrate synthase (CS), succinate-dehydrogenase (SDH) and ATPase activity of complex V.

Female BALB/c mice were acquired from the Laboratory Animal Breeding Center, Oswaldo Cruz Institute Foundation, Brazil. At eight weeks of age, with an average weight of 18 g, they were subjected to heart function analyses by echocardiography (Echo), electrocardiography (ECG) and ergospirometry (Ergo) prior to MeHg administration. Animals subsequently received a daily dose of MilliQ ultrapure water (MILLI-Q DIRECT-Q5, Millipore) (20 µL/g body weight; control group) or 5 mg.kg^−1^ methylmercury chloride (Methylmercury(II) chloride PESTANAL^®^ Merck analytical standard, purity > 97.8%) by gavage over 14 days (cumulative dose of 70 mg.kg^−1^; MeHg70 group). MeHg was diluted in MilliQ ultrapure water (2.5 mg/10 mL) and gently shaken for 30 min in a glass vial protected from light before gavage (20 µL/g). Then, both groups were subjected to the same heart function analyses. Body weight, survival rate, blood haemoglobin (Hb) content and MeHg concentration in heart tissue were also measured. All the animals were euthanised by cervical dislocation preceded by anaesthesia with 100 mg.kg^−1^ ketamine and 10 mg.kg^−1^ xylazine. Heart tissue samples were collected for high-resolution oxygraphy (Oxy), as well as for analyses of citrate synthase activity (CS), succinate dehydrogenase activity (SDH) and ATPase activity of complex V. mRNA for relative quantification by qPCR was isolated (see Figure 1). All reagents used in these assays were purchased from Sigma–Aldrich (St. Louis, MO, USA) unless otherwise indicated. They were individually identified and maintained under a 12 h light/dark cycle at 25 °C. They were also provided free access to filtered water and a standard rodent diet (Nuvilab, from Quimtia, PR, Brazil) at a local animal facility (Institute of Biophysics Carlos Chagas Filho—IBCCF, UFRJ, Rio de Janeiro, Brazil). This study was approved by the Ethics Committee on the Use of Animals of the Health Sciences Centre of the Federal University of Rio de Janeiro, Brazil (CEUA/CCS/UFRJ, CONCEA registration # 01200.001568/2013.87, approved protocol IBCCF 027/14). The animals received care in compliance with the “Principles of Laboratory Animal Care” formulated by the National Society for Medical Research and the “Guide for the Care and Use of Laboratory Animals” prepared by the National Academy of Sciences, USA, and the National Council for Controlling Animal Experimentation, Ministry of Science, Technology and Innovation (CONCEA/MCTI), Brazil.

### 2.2. Gas Chromatography–Atomic Fluorescence Spectrometry

Heart tissue samples from both groups were obtained after euthanasia under anaesthesia (100 mg.kg^−1^ of ketamine and 10 mg.kg^−1^ of xylazine). The method described by Liang et al. [32] was used to measure the MeHg concentration. After euthanasia, a known amount of hearts from both groups (between 20 and 100 mg) was freeze-dried in a rotary vacuum concentrator (AVC 2-25 CD plus CT 02-50 SR Christ, Analitica). These samples were transferred to polytetrafluoroethylene tubes, and 3.0 mL of a 25% (*w*/*v*) KOH/methanol solution was added for MeHg extraction in an oven at 70 °C (Nova Instruments, Model NI 1512, São Paulo, Brazil) for 5 h with gentle stirring every hour. The samples were kept in the dark to avoid possible photochemical MeHg degradation. Subsequently, ethylation was carried out using 300 µL of 2 mol.L^−1^ acetate buffer (pH 4.5), followed by the addition of 30 µL of sample and 50 µL of sodium tetraethylborate (1% *w*/*v*) following the method reported by Taylor et al. [33]. Ethylmethylmercury was purged onto tenax traps for 5 min with Hg-free argon and thermally desorbed (~200 °C) onto an isothermal GC column at 36.6 °C. Finally, Hg species were reduced to Hg^0^ by heating in the pyrolysis unit and then detected by cold vapor atomic fluorescence spectrophotometer (MERX-TM automated system from Brooks Rand Labs, Seattle, WA, USA) equipped with an autosampler [33]. The wavelength of 253.7 nm was used to measure the mercury fluorescence signal. The precision and accuracy of the MeHg determinations were ensured using triplicate analyses of heart samples. The certified reference materials are NIST-2976 and DOLT-2. The certificate value for NIST-2976 is 28.09 µg.kg^−1^, and we found 27.94 ± 1.72 µg.kg^−1^, corresponding to a recovery of 99%. The certificate value for DOLT-2 is 693 µg.kg^−1^, and we detected 704 ± 11 µg.kg^−1^, corresponding to a recovery of 101%. All MeHg results of the analysed samples are presented in mg.kg^−1^.

### 2.3. RNA Isolation and Real-Time Quantitative PCR (RT-qPCR)

Immediately after euthanasia, animals’ hearts were collected, and the tip of each heart (samples with about 20 mg of tissue) was cut off with a scalpel and placed in ice-cold TRIzol^®^. Total RNA was isolated according to the manufacturer’s instructions. The RNA concentration was quantified, and the purity was assessed via a Nanodrop Lite spectrophotometer (Thermo Scientific, Waltham, MA, USA). An amount of 10–15 µg of total RNA was obtained per sample. cDNA was synthesised from 1 µg of total RNA using the High-Capacity Reverse Transcription Kit with RNase Inhibitor (Thermo Fisher Scientific, Waltham, MA, USA) according to the manufacturer’s instructions. Real-time quantitative PCR (RT-qPCR) was performed via GoTaq^®^ qPCR Master Mix (Promega Corporation, WI, USA) on an Applied Biosystems 7500 Fast Real-Time PCR System. The mRNA expression of atrial natriuretic peptide (Nppa gene, ENSMUSG00000041616) was measured using primers with the following sequences: 5′-TGAAAAGCAAACTGAGGGCT and 5′-GGATCTTTTGCGATCTGCTC (166 bp). Glyceraldehyde 6-phosphate dehydrogenase (Gapdh gene, ENSMUSG00000057666) was used as the housekeeping gene [5′-CAGCCTCCCGCTTCGCTCTC and 5′-CCAGCATAACCCGCGGACCA (76 bp)]. The primers were designed via the Ensemble Genome Browser (Ensemble.org). Relative cDNA expression was calculated via the comparative cycle threshold method. The expression of the Nppa gene is expressed relative to that of the Gapdh gene.

### 2.4. Haemoglobin and Cholesterol Measurements

Blood samples were collected from the retro-orbital venous plexus of anaesthetised mice before euthanasia. Blood samples with 0.2 mL were transferred to vacutainer tubes with 18 mg of K_2_EDTA (ethylenediaminetetraacetic acid; EDTA.k2 0.5 mL, Guangzhou Improve Medical Instruments Co., Ltd., Guangzhou, China) and maintained at 4 °C until the measurement of haemoglobin levels. The haemoglobin quantification was carried out via photometry on an automated analyser (Micros 60 ABX, Horiba Medical, Irvine, CA, USA). Cholesterol concentration was measured in serum samples. Blood samples of 0.2 mL were left at room temperature in glass tubes (with serum separating gel, Guangzhou Improve Medical Instruments Co., Ltd., Guangzhou, China) to allow for clot formation and centrifuged (10,000× *g* for 5 min). The cholesterol quantification was carried out via the colorimetric enzymatic method (100/280-200 kit; VIDA Biotecnologia Ltd., Minas Gerais, Brazil), with wavelength reading in a spectrophotometer at 500 nm.

### 2.5. Ergospirometry

The animals were subjected to a treadmill ergometer test using a system from AVS Projetos Ltd. (Rio de Janeiro, Brazil) coupled to a gas analyser for O_2_ consumption measurements (VO_2_) before and after MeHg or MilliQ ultrapure water administration (modified from Martinez et al. [34]). The day before each test, the animals were familiarised with the device (10 min for environmental recognition followed by 5 min of walking at 6 m.min^−1^). The test protocol consisted of 10 min of recognition followed by running at 6 m.min^−1^ for one min. Every minute, the velocity was increased by 2 m.min^−1^ until exhaustion, which was defined as four extended contacts with the shock plate (0.2 mA) behind the treadmill. An elevation angle of 18° was maintained throughout the duration of the experiment. The amount of time spent walking required to reach exhaustion (TTE, time of tolerance to effort) and the VO_2_ (maximal oxygen consumption related to effort) were evaluated. The software AQCAD 2.4.8 (AVS Projetos, Rio de Janeiro, Brazil) was used for all analyses.

### 2.6. Electrocardiography

The animals were anaesthetised via an intraperitoneal injection of 10 mg.kg^−1^ xylazine and 100 mg.kg^−1^ ketamine and placed on a platform in the prone position. Steel electrodes were implanted in subcutaneous tissue. The protocol was adapted from Martinez et al., 2015 [34]. ECG records (bipolar lead DI) were acquired from awake and anaesthetised animals using the PowerLab 400 System (AD Instruments, Castle Hill, Australia). Tracings were recorded and archived for offline analysis using LabChart 7 software. The following parameters were evaluated: heart rate (HR), P-R interval duration (atrioventricular electric conduction), P wave duration and height (atrial depolarisation pattern, displaying the origin of the stimulus, whether in the sinus node or ectopic pacemaker), QRS complex duration (ventricular depolarisation pattern) and corrected QT interval (QTc; indicates whether ventricular repolarisation is homogeneous). Repolarisation abnormalities, such as P-R and ST depression or elevation, and arrhythmias were also detected.

### 2.7. Echocardiography

Echocardiographic exams (blinded protocol) were performed by an investigator proficient in small animal echocardiography using a 10 MHz sectorial transducer (MyLab equipment, Esaote Inc., Genoa, Italy). The protocol was modified from Silva et al. [35], and the exams were performed on anaesthetised mice (10 mg.kg^−1^ xylazine and 100 mg.kg^−1^ ketamine). The following measurements were obtained: heart rate (HR), left ventricle anterior wall diastolic thickness (LVAWDT), left ventricle end-diastolic diameter (LVEDD), left ventricle end-systolic diameter (LVESD), left ventricle ejection fraction (EF) calculated via the Simpson modified method [36] and cardiac output (CO = stroke volume × heart rate). The cardiac output is a measurement of the amount of blood pumped by the left ventricle in one minute. Left ventricle (LV) diastolic function was evaluated using the E/A wave velocity ratio and the isovolumetric relaxation time (IVRT) [36].

### 2.8. Mitochondrial Analysis

#### 2.8.1. Citrate Synthase (CS) Activity Assay

Citrate synthase activity, which is considered a validated marker of mitochondrial density in striated skeletal muscle [37], was performed according to the method described by Cerqueira et al. [38], with modifications. Briefly, left ventricle (LV) fragments were homogenised using a T50 Ultra-Turrax^®^ disperser (Ika, Germany) in cold lysis buffer (50 mM sodium phosphate, pH 7.4; 10% glycerol; 1% octyl phenol ethoxylate; 10 mM sodium orthovanadate; 10 mM sodium fluoride; and 10 mM sodium pyrophosphate supplemented with a protease inhibitor cocktail cOmplete^®^ Roche, Basel, Switzerland). After 30 min on ice, the sample lysates were centrifuged (12,000× *g*, 20 min, 4 °C), and the resulting supernatants were collected. Protein was quantified using the Bradford method [39], modified from the methods of the Bio-Rad Protein Assay Kit I. A reaction mixture with 20 mM Tris-HCl (pH 8.0), 0.42 mM acetyl-coenzyme A, 0.1 mM DTNB [5′,5′-dithiobis (2-nitrobenzoic acid)] and 5 µg of total protein was incubated at 37 °C for 5 min. Oxaloacetate (5 mM) was used to initiate the reaction. The reduction in DTNB by citrate synthase was measured spectrophotometrically for 10 min at 412 nm (molar extinction coefficient = 13.6 mM^−1^.cm^−1^). The results are expressed as units.mg^−1^.min^−1^.

#### 2.8.2. Succinate Dehydrogenase (SDH) Activity Colorimetric Assay

SDH activity was measured in heart tissue homogenate. The LV fragment was homogenised in the cold lysis buffer. The samples were centrifuged at 12,000× *g* at 4 °C for 10 min. Protein in the supernatant was quantified, and 25 µg was used in a final volume of 200 µL in each microplate well. The samples were processed in triplicate with 4 mM sodium azide, 200 µM phenazine methosulfate, 20 mM phosphate buffer (pH 7.5) and 0.1% Triton X-100. The reaction was initiated by the addition of 10 mM succinate. The kinetics of the reduction in 2,6-dinitrophenol-indophenol [50 µM (DCIP, Riedel de Haën)] sensitive to 20 mM malonate were subsequently measured for 60 min at 600 nm (modified from Schwall et al. [40]). SDH activity is expressed in the U.mg^−1^ protein.CS^−1^.

#### 2.8.3. ATPase Activity of Complex V

The ATPase activity of complex V was measured in a membrane-enriched fraction of heart tissue. Left ventricle samples were homogenised in extraction buffer (20 mM Tris-HCl pH 7.4, 1 mM EDTA, 50 mM KCl, 0.1% Triton X-100, 250 mM saccharose and cOmplete^®^Roche Protease Inhibitor Complex). Briefly, the samples were centrifuged at 600× *g* for 10 min, and the connective tissue was discarded. The supernatants were again centrifuged for 10 min at 12,000× *g*, and the pellets were subsequently resuspended in an extraction buffer. After protein quantification, 0.1 mg/mL protein was used in each reaction. The assay was performed in triplicate at 37 °C. Thapsigargin (0.1 mM; SERCA inhibitor) and ouabain (1 mM; sodium–potassium ATPase inhibitor) were added to the reaction buffer (25 mM Tris-HCl, 1 mM EDTA, 10 mM MgCl_2_, 50 mM KCl). We subtracted 5 mM azide-sensitive hydrolysis to exclude nonspecific ATPase activity. The reaction was initiated by 5 mM [γ-^32^P] ATP and stopped after 40 min with 0.1 M HCl. Next, activated charcoal in 0.1 M HCl was added. After centrifuging for 6 min at 13,500× *g*, the radioactive [γ-^32^P] P_i_ in the supernatant was measured via liquid scintillation.

#### 2.8.4. Mitochondrial Oxygen Consumption

Mitochondrial oxygen consumption (mtVO_2_) related to the mitochondrial electron-transport system (ETS) was measured in permeabilised heart fibres using three different protocols [41] in an Oroboros Oxygraph-O2K system (Oroboros Instruments, Innsbruck, Austria). Analyses were performed at 37 °C using MiR05 buffer.

A slice of the heart close to the apex was cut with a sharp scalpel blade immediately after the animal died. The samples of about 5 mg of tissue were preserved in ice-cold relaxing BIOPS solution (2.77 mM CaK_2_EGTA, 7.23 mM K_2_EDTA, 5.77 mM Na_2_ATP, 6.56 mM MgCl_2_·6H_2_O, 20 mM taurine, 15 mM Na_2_ phosphocreatine, 20 mM imidazole, 0.5 mM dithiothreitol and 50 mM MES; pH 7.1) for no more than one hour. Fiber bundles were mechanically separated using small forceps. Next, they were permeabilised with saponin (25 µg/mL) for 15 min with gentle shaking in ice-cold BIOPS [42]. The samples were then washed twice with cold MiR05 respiration medium (0.5 mM EGTA, 3 mM MgCl_2_, 60 mM K^+^-lactobionate, 20 mM taurine, 10 mM KH_2_PO_4_, 1 mg/mL bovine serum albumin and 20 mM HEPES; pH 7.1).

To obtain an overview of the functional interface between ETS and the oxidative phosphorylation system (OXPHOS), the following multisubstrate sequence was used: 5.0 mM pyruvate/2 mM malate (PM, complex I substrates); 2 mM ADP (ATP synthase complex V substrate); 10 μM cytochrome C (cytochrome C, to test the mitochondrial internal membrane integrity); 10 mM succinate (Succ, complex II substrate); 1 µg/mL oligomycin (Omy, complex V inhibitor); 0.5 μM carbonyl cyanide 4-(trifluoromethoxy) phenylhydrazone (FCCP, an uncoupling agent); 1.0 μM rotenone (Rot, complex I inhibitor); 10 mM malonate (Malo, complex II inhibitor); and 2.0 mM KCN (finally inhibiting complex IV).

Respiratory capacity was determined by the difference between the peak mtVO_2_ (after FCCP addition in the presence of substrates and ADP) and the basal mtVO_2_. This measure represents the ETS capacity without restriction of the proton motive force (ΔΨm). The dissipative non-phosphorylating state (leak) was induced by the addition of oligomycin in the presence of complex I and II substrates. Oxygen consumption related to ATP synthesis was calculated by subtracting the oxygen consumption level achieved after oligomycin addition during state 3. The respiratory control ratio was calculated as the oxygen flux in the presence of ADP plus complex I and II substrates divided by the oxygen flux in the presence of oligomycin.

A second protocol was performed to investigate the threshold for mitochondrial permeability transition pore (mPTP) opening, with the sequential addition of CaCl_2_ to medium containing permeabilised heart fibres (modified from Anderson et al. [43]). First, the samples were incubated for 30 min in the presence of 3 mg.mL^−1^ collagenase type III (Sigma) in cold BIOPS solution. The samples were then subjected to the permeabilisation procedure previously described, followed by two rinses with MiR05 buffer. MiR05 respiration buffer was supplemented with 100 µM ADP, 5 mM glucose, 1 U/mL hexokinase, 10 µM thapsigargin (SERCA inhibitor), 10 µM EGTA, 5.0 mM pyruvate, 0.5 mM malate and 10 mM glutamate. CaCl_2_ was sequentially added (100 µM pulses) to induce a sudden increase in oxygen consumption (defined as the mPTP threshold) up to a cumulative CaCl_2_ dose of 2 mM.

Finally, carboxyatractyloside was titrated over the range of 5 to 1000 nM, and 5.0 mM pyruvate, 0.5 mM malate, 2.0 mM ADP and 10 mM succinate were used as substrates for the respiratory complexes.

The mtVO_2_ values obtained via the multisubstrate protocol, the ATPase activity assay, and titrations with carboxyatractyloside and calcium for ANT and mPTP threshold analyses were normalised to citrate synthase activity and expressed in [pmol.mg^−1^.s^−1^]/CS/g wet tissue.

#### 2.8.5. Western Blot Analysis of the Heart Tissue

Proteins of control and MeHg70 heart tissues (10 µg sample/lane, N = 3 animals per group) were separated via 15% SDS polyacrylamide gel electrophoresis and transferred to a nitrocellulose membrane by electroblotting for 90 min at 350 mA and 4 °C in a Mini-Protean Tetra System from Bio-Rad. The membrane was blocked for 120 min with 5% non-fat dry milk in TBS + 0.05% Tween 20 (TBS-T) and incubated overnight at 4 °C with primary antibodies against adenine nucleotide translocase (ANT) (1:4000 dilution; Santa Cruz Biotechnology sc9299, Santa Cruz Biotechnology, Dallas, TX, USA), OxPhos Rodent WB antibody (1:400 dilution; Invitrogen #45-8099) or/and vinculin (1:2000 dilution; Sigma–Aldrich V9131), which was used as a loading control, diluted in 5% non-fat milk. After five washes (10 min each), the membranes were incubated with peroxidase-conjugated secondary antibodies diluted in TBS-T for 1 h. After another five (10 min each) washing steps, the membranes were incubated with a peroxidase substrate (SuperSignal West Femto/Chemiluminescent Western Blot Detection, Thermo Scientific, USA). Protein bands were visualised by chemiluminescence using the imageQuant4000LAS system and quantified via densitometry 358 using the ImageJ software program. Complex I, II, III, IV and V subunits and proteins have the following molecular weights: C I, 20 kDa; C II, 30 kDa; C III, 48 kDa; C IV, 40 kDa; C V 55 kDa, ANT, 33 kDa; and vinculin, 116 kDa [44].

### 2.9. Statistics

The results are expressed as the average ± standard error. Student’s t-test was used to compare data between the two groups. For three or more groups, data were compared using one-way or repeated-measures two-way ANOVA. Post hoc Bonferroni correction was used for multiple comparisons. The data were organised in Excel (Microsoft Corporation, Redmond, WA, USA) and analysed using GraphPad Prism 5.0 (GraphPad Prism, Redmond, WA, USA). * *p* < 0.05, ** *p* < 0.01 and *** *p* < 0.001 were used to indicate significant differences between the MeHg70 group and the control group.

## 3. Results

### 3.1. MeHg Compromised the Viability of the Mice

In an independent experiment, the general condition of 25 mice from the MeHg70 group and 25 mice from the control group were evaluated. Body weight was significantly lower for animals from the MeHg70 group than for those in the control group (N = 25 per group); mice in the control group presented a weight gain ratio of approximately 10%, which is typical for their age (Table 1). Our results are consistent with those reported by other authors, i.e., oral gavage of 84 mg.kg^−1^ of MeHg for 28 days [45] or 54 mg.kg^−1^ for 13 weeks [46] resulted in weight loss in rats, indicating that decreased body weight is a sign of MeHg intoxication.

A reduced survival rate was also observed in the MeHg70 group (46.4%) when compared to the control group (95.23%) (Table 1). Only one animal from the control group died from an unknown cause.

The concentration of MeHg observed in the heart of animals from the MeHg70 group was 23.85 ± 2.62 mg.kg^−1^, an amount lower than that detected in skeletal muscle (308.1 ± 35.4 mg.kg^−1^) as described in previous work by our group, using a similar protocol [47]. Rodrigues et al. (2010) [48] also found medium levels of MeHg in rats’ hearts 5 days after receiving, via gavage, one dose of 0.5 mg MeHg.kg^−1^. A small amount of this compound was detected in animals’ blood on the 4th day. They proposed that the high mobility of methylmercury from blood to tissues occurred due to the formation of thiol complexes with cysteine and homocysteine, which are readily transported across cell membranes to tissues via L-type large neutral amino acid transporters (LATs). In the cells, they bind to intracellular glutathione, whose concentration is in the mM range in most mammalian cells [49]. This issue was very well explored in a recent review [50], highlighting metallomic studies. They emphasise that the integration of bioinorganic chemistry events, such as intoxication by MeHg, in the blood plasma–red blood cells–organ system is critical to causally link human exposure to toxic metal species with diseases, such as cardiac damage shown in this manuscript.

**Figure 2:**
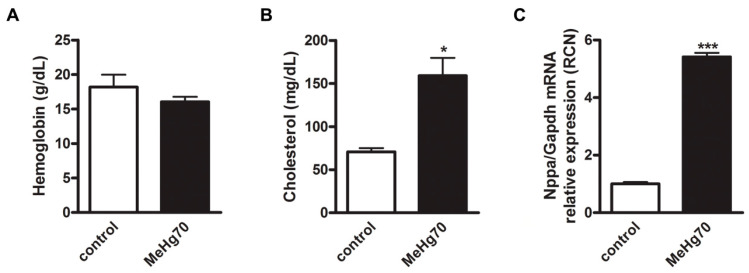
(**A**) Blood haemoglobin (g/dL, N = 5): (**B**) Serum total cholesterol (mg/dL, N = 5); (**C**) Atrial natriuretic peptide mRNA expression (nPPA gene) was measured in the heart tissue of mice in the MeHg70 and control groups (normalised to glyceraldehyde-3-phosphate dehydrogenase (GAPDH) mRNA expression), (N = 4). The bars represent means ± SEs. * *p* < 0.05 and *** *p* < 0.001.

**Figure 3:**
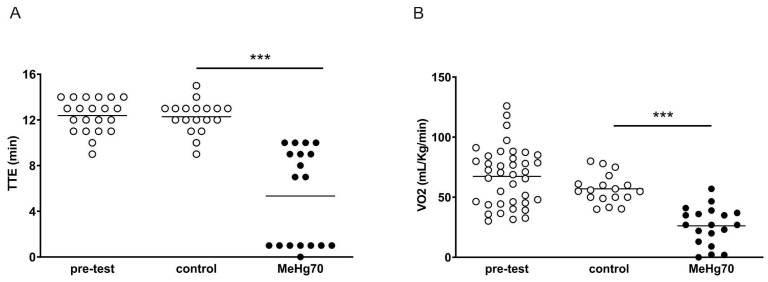
Ergospirometry. The pretest results and retest results for the MeHg70 group and the control group are shown: (**A**) Time of tolerance to effort (TTE). N = 21 (pretest), 18 (control group), 18 (MeHg70 group); (**B**) Peak oxygen consumption (VO_2_). N = 39 (pretest), 18 (control group), 19 (MeHg70 group). *** *p* < 0.001.

No differences in haemoglobin levels were detected between the MeHg70 group and the control group (Figure 2A). Therefore, the reduction in VO_2_ in animals in the MeHg70 group, as evidenced by the ergospirometry results, which is shown later in Figure 3, may not be related to anaemia. We propose that this may be related to a reduction in cardiac reserve resulting from direct cardiotoxicity. In addition, other mechanisms may be involved. In 2020, Piscopo et al. [51] reported that in human erythrocytes in vitro, HgCl_2_ induces the tetramerisation of haemoglobin. This can modify the haemoglobin dissociation curve and impair O_2_ delivery to tissues.

Additionally, total serum cholesterol levels were greater in the MeHg70-treated animals than in the control animals (Figure 2B). As expected, blood serum samples from this group had a “milky” appearance. This finding corroborates results previously described in human population studies that suggest a causal relationship between mercury exposure and dyslipidemia [52,53,54], which is a well-known risk factor for cardiovascular diseases [55]. Figure 2 also shows a 4-fold increase in atrial natriuretic peptide (ANP) mRNA expression, as detected by qPCR, in the MeHg70-treated animals (Figure 2C). This result indicates the presence of left ventricle (LV) systolic stress, which was confirmed by the echocardiography results (Table 3). LV systolic stress is the initial result of alterations in the ability of the heart to eject blood to the circulatory system; its detection is crucial for diagnosis and more efficient treatment. It occurs prior to the clear deterioration of heart function, which is sometimes irreversible.

### 3.2. MeHg Administration Worsens Ergospirometry Performance

Compared with mice in the control group, mice in the MeHg70 group presented a shorter tolerance time for running in the treadmill test (TTE) (MeHg70: 5.3 ± 0.9 vs. 12.2 ± 0.3 min, *p* < 0.0001) (Figure 3A). Notably, the MeHg70-treated animals displayed a bimodal TTE pattern, in which half of the animals ran for approximately 30% less time than the controls, and the rest were hardly able to run at all. Animals in the MeHg70 group also presented lower VO_2_ values than those in the control group (26.2 ± 3.6 vs. 56.9 ± 2.8 mL.kg^−1^.min^−1^, *p* < 0.0001; Figure 3B). There was no anaemia or significant reduction in cardiac output (amount of blood pumped by the left ventricle in one minute), only a nonsignificant slight trend, in the animals in the MeHg70 group. Our group previously demonstrated in 2021 that the same dose of methylmercury induces muscle atrophy via an increase in proteolysis and a decrease in protein synthesis in the gastrocnemius and soleus muscles of female mice [47]. Therefore, skeletal muscle damage may also have contributed to the worse performance of the MeHg70 group.

### 3.3. MeHg Altered Heart Automaticity, Atrioventricular Conduction and Ventricular Repolarisation

The electrocardiography results obtained from anaesthetised and non-anaesthetised animals are shown in Table 2. Compared with the control animals, the MeHg70-treated animals presented a striking reduction in the heart rate of the awake animals. This finding indicates heart autonomic system modulation, with an imbalance favouring parasympathetic tone. This difference is quite notable, considering that the usual response to stress-systolic conditions is to accelerate the heart rate to maintain cardiac output (cardiac output = stroke volume x heart rate) even with a reduced ejection volume of the left ventricle. The occurrence of autonomic changes induced by MeHg has already been described, although its clinical significance is uncertain [56]. Nonetheless, in many cardiomyopathies, autonomic imbalance is associated with a poor prognosis [57,58]. Atrial electrical activation (p wave characteristics) remained unaltered.

The sinus node is the heart’s natural pacemaker. The duration of the P-R interval represents the time taken for the electrical impulse to travel from the sinus node to the atrioventricular node, from where it is passed on to the ventricles. The greater the P-R interval, the slower the atrioventricular conduction is. We detected a slowed atrioventricular conduction (P-R enlargement) in anaesthetised animals from the MeHg70 group. In non-anaesthetised animals, the shortening of the P-R interval secondary to tachycardia made it difficult to highlight differences between the groups. Both heart rate and atrioventricular conduction are closely related to the pacemaker cells of the heart, not cardiomyocytes, which specialise in blood pumping.

Slower intraventricular electrical conduction (depolarisation) increases the duration of the QRS complex. In a similar way, slower ventricular repolarisation increases the QTc duration. The animals in the MeHg70 group exhibited slowing of ventricular depolarisation (QRS) and repolarisation (QTc). QRS patterns are also determined by pacemaker cells and are less influenced by the heart rate range than the P-R interval. The QRS enlargement observed in awake animals in the MeHg70 group was not accompanied by a change in the electrical axis of the complexes, indicating that it was the result of slight and diffuse hypoactivity of pacemaker cells in the ventricles that did not produce a sharp cardiac bundle blockage, under the tested conditions. It is possible that, in accordance with the natural occurrence of these events, a longer period of observation could reveal typical heart blockage.

An increase in the QTc interval was noticed in the anaesthetised and awake animals from the MeHg70 group, compared with the control group in both conditions, which indicates persistent repolarisation heterogeneity (long QTc). In this setting, extrasystoles (early beats) may cause waves of electrical activation in part of the cardiomyocytes, which are already repolarised, while others are still refractory to electrical stimulation. This is the substrate for re-entry, which in the ventricles can trigger ominous arrhythmias such as torsades de pointes, which can cause sudden death [59]. It has already been reported in humans poisoned with fungicides containing ethyl mercury [19,20].

**Figure 4:**
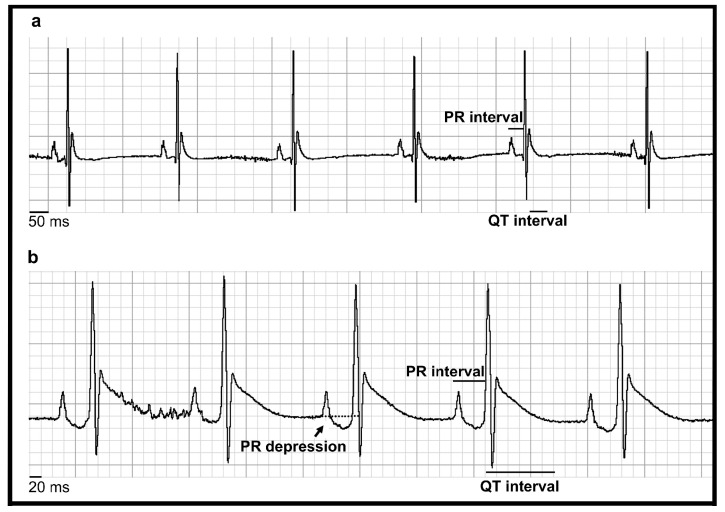
Electrocardiography results obtained in anaesthetised animals: (**a**) Representative trace from one control animal. The P-R and QT intervals are highlighted. (**b**) Representative trace from one animal in the MeHg70 group. The P-R interval, QRS complex duration and QTc were prolonged. There was also P-R segment depression.

In Figure 4, a representative ECG of one animal from the control group is compared with an ECG from one animal from the MeHg70 group, in which impaired ventricular depolarisation and repolarisation were observed. The animal whose ECG is shown was one of the animals from the MeHg70 group that displayed P-R interval slowing, characterised by atrioventricular conduction delay. In addition, P-R depression was observed. These P-R changes are likely the result of myocardial inflammation, as described in human myopericarditis [60,61].

### 3.4. MeHg Poisoning Induced Left Ventricle Stress

Table 3 presents the echocardiography results. Compared with that in mice in the control group, the LV anterior wall diastolic thickness (LVAWDT) in mice in the MeHg70-treated group was lower (*p* < 0.05), indicating a reduction in the left ventricle mass. Protein homeostasis is essential for cardiac tissue viability, whose contractile cells have little capacity to renew. Imbalances in protein quality control systems have been described as relevant mechanisms in the evolution of several heart diseases, such as those related to diabetes, hypertension and ischemia [62,63].

In 2021, our group described a reduction in muscle mass and myosin-heavy chain content in the soleus and gastrocnemius muscle of mice intoxicated with the same dose of MeHg as used in this work. In addition, these animals showed unfavourable modulation in protein quality control systems, such as the ubiquitin–proteasome system, with a predominance of proteolysis [47]. It is possible that similar events were occurring in the cardiac striated muscle. As far as we know, there is a lack of studies addressing heart muscle mass loss in MeHg intoxication.

Additionally, although there was no difference in the diastolic diameter between the groups, there was an increase in the systolic diameter of the LV in the MeHg70 group. This increase caused a statistically significant reduction in the ejection fraction in the MeHg70 group relative to that in the control group (*p* < 0.01). These two findings are often observed in the early phase of cardiomyopathies before overt systolic dysfunction.

The atrial wall is very thin, and when there is an increase in LV pressure, dilation of the atrial cavity usually occurs. A reduction in the aorta/left atrium ratio, which is an indirect measure of LV systolic stress, was also detected in the MeHg70 group (*p* < 0.05). Nevertheless, obvious heart failure at rest was not observed, as cardiac output was not significantly reduced in the MeHg70 group. We propose that animals from the MeHg70 group have low LV contractile reserve (ability to increase cardiac output), which causes the worst performance in ergospirometry.

The heart consumes a large amount of ATP, approximately 70% of which is used for contractile function. Furthermore, mitochondrial oxidative metabolism is responsible for almost all the ATP consumed by the heart [64]. Based on these data, analyses of oxygen consumption in cardiac tissue are very important complements to the study of cardiac function.

The heart rate of animals under anaesthesia was also assessed. No differences that could interfere with systolic performance were observed. Diastolic function was evaluated by the isovolumetric relaxation time (IVRT) and the E wave/A wave ratio because advanced diastolic dysfunction may cause signs of heart failure even with preserved left ventricular systolic function [65]. No differences between the groups were detected (Table 3), and this can be related to the low sensitivity of the available parameters, which were developed for humans and are not as informative in the presence of a heart rate greater than 100 BPM.

### 3.5. MeHg Reduced Mitochondrial Respiration without Affecting the Mitochondrial Content

**Figure 5:**
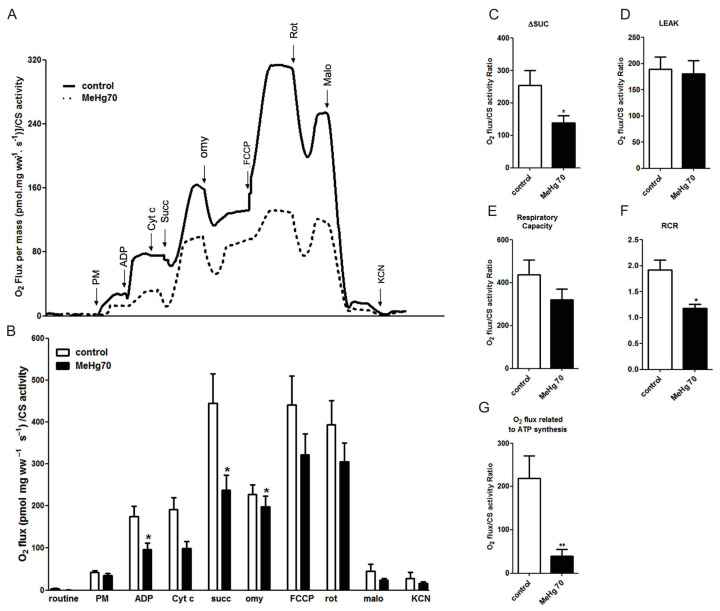
High-resolution oxygraphy of permeabilised heart fibres: (**A**) Representative traces of one animal from the control group (continuous line) and the MeHg70 group (dotted line). The arrows represent the administration of each substrate. PM = pyruvate + malate; ADP = adenosine diphosphate; Cyt c = cytochrome c; succ = succinate; omy = oligomycin; FCCP = p-trifluoromethoxyphenylhydrazone; rot = rotenone; malo = malonate; and KCN = potassium cyanide. (**B**) High-resolution oxygraphy results for the MeHg70 group (black bars) and control group (white bars). The bars represent means ± SEs. O_2_ flux is plotted in the routine state (without exogenous substrates), and after the addition of pyruvate + malate (PM), ADP, cytochrome c (Cyt c), succinate (succ), oligomycin (omy), p-trifluoromethoxyphenylhydrazone (FCCP), rotenone (rot), malonate (malo), and potassium cyanide (KCN); ww = wet weight. (N = 9) * *p* < 0.05. (**C**–**G**) High-resolution oxygraphy of permeabilised heart fibres from the MeHg70 and control groups normalised to mass and citrate synthase activity. (**C**) mtVO_2_ increased after the addition of succinate. (**D**) Leak state. (**E**) Respiratory capacity. (**F**) RCR = respiratory control ratio. (**G**) mtVO_2_ is related to ATP synthesis. The bars represent means ± SEs. N = 9, * *p* < 0.05, ** *p* < 0.01 when data from animals from the MeHg70 group were compared to animals from the control group.

For a general assessment of the state of the electron-transport system (ETS) and the oxidative phosphorylation system (OXPHOS), a multisubstrate protocol was carried out [41]. Figure 5A shows representative traces of the oxygen flux by high-resolution oxygraphy of permeabilised heart fibres from the control (continuous line) and MeHg70 (dotted line) groups. The averaged data of each group (N = 9) are displayed in Figure 5B. The addition of complex I substrates (pyruvate and malate) induced a similar response in both groups (Figure 5B). However, when ADP was added, mtVO_2_ did not increase in the MeHg70 group animals as it did in the control group. Oligomycin administration reduces oxygen consumption related to complex V. Attenuation of this response to oligomycin indicates a reduction in the activity of complex V. In a complementary way, the mtVO_2_ reduction induced by oligomycin was attenuated in sample fibres from mice in the MeHg70 group when compared with sample fibres from mice in the control group. The response to succinate administration (triggering parallel electron input from complexes I [NADH dehydrogenase] + II) was also reduced in the MeHg70 group when compared with the response observed in mice from the control group.

These results are highlighted in Figure 5C–G. The mtVO_2_ range after succinate addition was 50% lower in the MeHg70 group than in the control group (*p* < 0.05, Figure 5C). The leak state was similar between the two groups, indicating that there was no change in the permeability of the internal mitochondrial membrane to protons (Figure 5D). The respiratory capacity of the MeHg70 group tended to decrease but did not reach statistical significance, and this lack of difference relative to that of the control group provides evidence of a reasonably preserved electron transport process (Figure 5E). The respiratory control ratio was reduced by 30% in the MeHg70 group relative to the control group, indicating OXPHOS impairment (Figure 5F). The level of oxygen consumption related to ATP was also reduced by a factor of four (Figure 5G), indicating functional deficiencies among OXPHOS components.

These changes were not supported by alterations in citrate synthase enzyme activity (CS), which is also considered a marker of mitochondrial content, since heart homogenates from the mice in both groups presented similar CS activities (Table 4). Additionally, the activity of succinate dehydrogenase/CS and the ATPase activity of complex V did not significantly differ between the groups, although the activity of complex V in the MeHg70 group tended to be lower than that in the control group (Table 4).

The reduced response of permeabilised fibres to succinate administration in the MeHg70 group during the multisubstrate protocol, combined with the relatively preserved respiratory capacity (indicating a preserved STE) and the absence of a reduction in the enzymatic activity of succinate dehydrogenase, indicates that the defect that restricts oxygen consumption is in the ATP synthesis system (complex V, adenine nucleotide translocase (ANT) and the phosphate carrier).

**Figure 6:**
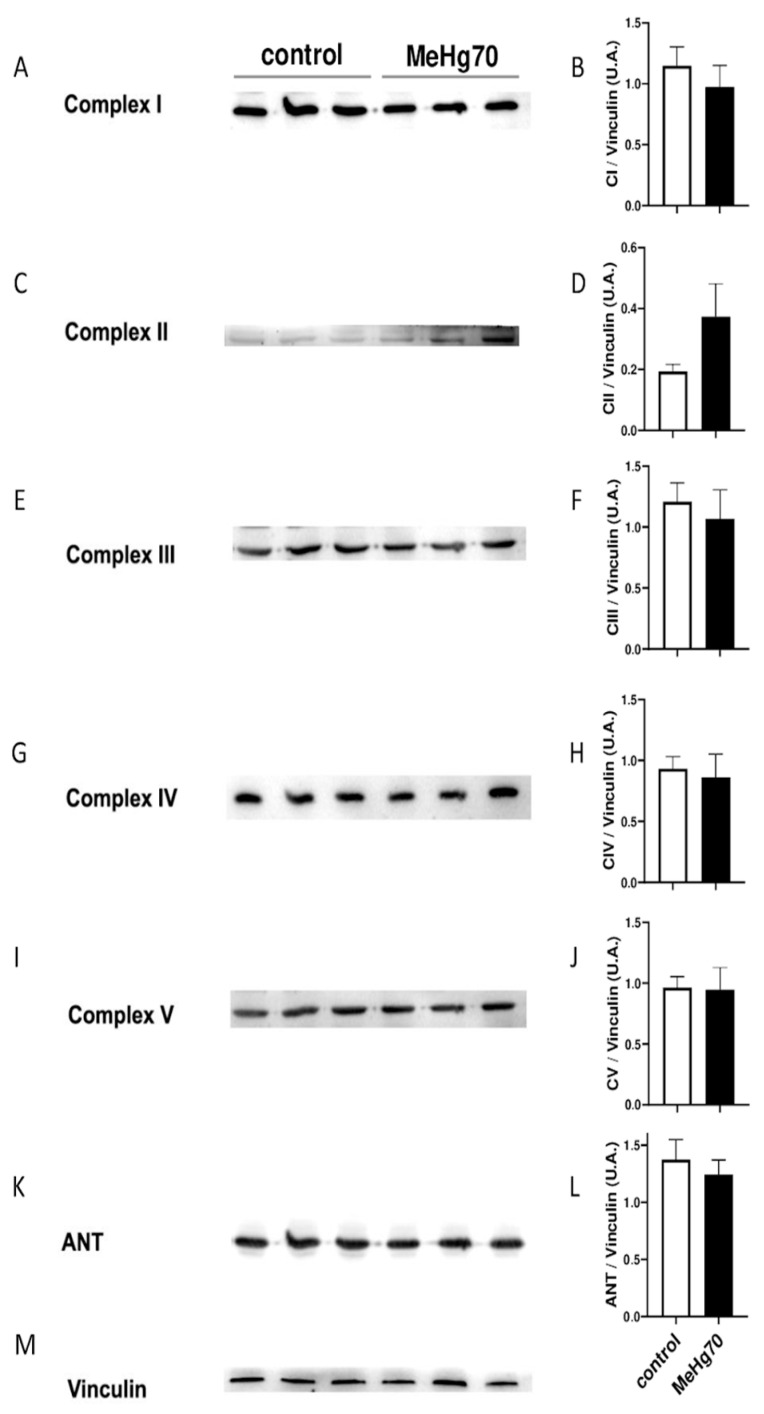
Protein expression in the membrane-enriched fraction of hearts from control and MeHg70 mice (N = 3 animals per group). Immunoblots for respiratory complexes (**A**,**C**,**E**,**G**,**I**), ANT (**K**) and the loading control (vinculin, (**M**)) are shown. Densitometric analysis of each protein normalised to vinculin (**B**,**D**,**F**,**H**,**J**,**L**). The graphs present means ± SEs.

When evaluated by immunoblotting, the amount of proteins from ETS subcomplexes, ATP synthase and ANT in heart homogenates of the MeHg70 group were not different from those in the samples from the control group (Figure 6). This result confirmed that both groups had similar mitochondrial contents.

Under our experimental conditions, the blunted mtVO_2_ increase after the addition of succinate seems to be more related to ATP synthesis system modulation than to a direct effect on succinate dehydrogenase, as there were no changes in enzyme activity or protein expression.

We hypothesise that a decrease in VO_2_ is associated mainly with a reduction in mitochondrial oxygen consumption in heart tissue. Additionally, a high concentration of MeHg accumulates in striated skeletal muscle, and this type of muscle is affected by MeHg [47]. Therefore, it is plausible that a similar disruption in OXPHOS may have occurred in the skeletal muscle of the MeHg70 group.

**Figure 7:**
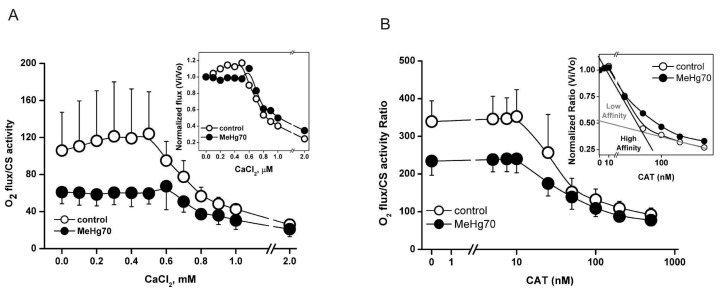
mtVO_2_/citrate synthase activity in permeabilised heart fibres of the MeHg70 and control groups normalised to heart fragment mass. The experiment was performed in the presence of pyruvate and malate (complex I substrates), succinate (complex II substrate) and ADP (**A**) Ca^2+^ titration for MPTP induction. (**B**) Carboxyatractyloside (Cat) titration to inhibit oxygen consumption related to complex V. The data represents ± SEs. The inset shows the changes normalised to the basal values of mtVO_2_ (N = 3).

Despite the absence of significant differences in the SDH and ATPase activity of complex V, the damage to mitochondrial oxidative phosphorylation in permeabilised heart fibres could be the result of dysfunction in one or more OXPHOS components. We tested sensitivity to the opening of the mitochondrial permeability transition pore (mPTP) by assessing the state of ETS and OXPHOS via calcium chloride titration. As exemplified in Figure 7A (dotted line), permeabilised fibres from the MeHg70 group displayed a lower initial mtVO_2_ in the presence of the complex I and II substrates (pyruvate, malate and succinate) and ADP than those from the control group (Figure 5C). Additionally, the MeHg70 fibres did not display an increase of 20% in oxygen consumption at Ca^2+^ concentrations of up to 200 µM, as observed in animals in the control group. Furthermore, a greater threshold for Ca^2+^-induced mPTP opening (600 µM vs. 500 µM) was observed in this group than in the control group.

Additionally, titrations with carboxyatractyloside (Cat; inhibitor of adenine nucleotide translocase—ANT) were performed in heart permeabilised fibres (Figure 7B). The mtVO_2_ in the presence of the complex I and II substrates plus ADP was lower in the MeHg70 group than in the control group, confirming the results obtained in the previous experiments (multisubstrate protocol in Figure 5 and Ca^2+^ titration in Figure 7A). Compared with the control group, the MeHg70 group also presented a modified response to Cat. This difference was characterised by a curve profile in which the higher affinity component (at Cat values between 30 and 40 nM) was attenuated, with the lower affinity component prevailing (approximately 500 nM).

The mitochondrial permeability transition pore (mPTP) is currently defined as a multiprotein complex of the inner and outer mitochondrial membrane that forms a nonselective channel under conditions of elevated matrix ion Ca^2+^ and oxidative stress. The precise molecular identity of the mPTP remains uncertain, but strong evidence suggests that the participation of mitochondrial complex V and adenine nucleotide translocase (ANT) in the pore-forming conformation, which is necessarily modulated by cyclophilin D, is present in the mitochondrial matrix [66]. Several mutations in SLC25A4, the gene that encodes ANT, are associated with primary mitochondriopathies, some of which present cardiomyopathy phenotypes characterised by fatigue and exercise intolerance, such as the Kearn–Sayre Syndrome (KSS) [63]. Cysteines 56, 159 and 256 of ANT are known to be responsive to thiol reagents and oxidative stress [64,65]. We hypothesised that the S-mercuration of some of these cysteines by MeHg could lead to nonfunctional ANTs, as demonstrated by the attenuation of the inhibition of O_2_ flux in response to Cat in MeHg70 mice. This situation could be like those faced by individuals with Kearn-Sayre Syndrome [63].

Similarly, Nesci et al. [66] used the mitochondrial fraction from swine hearts and reported that micromolar HgCl_2_ concentrations inhibited the respiration of NADH-energised mitochondria, whereas this effect did not occur when the substrate was succinate. They also reported that the activity of F1F0-ATPase decreased 100% in the presence of 100 µM Hg^2+^ and that this decrease was reversed by the addition of thiol-reducing agents. They suggested that Hg^2+^ interacts with the thiol residues of OXPHOS complexes and modulates their functionality by modifying the redox state of their thiol groups. Since an increase in Ca^2+^ concentrations in mitochondria is accompanied by mPTP opening and the participation of the mitochondrial F1Fo-ATPase in mPTP opening is transiently sustained, we recommend additional investigations to analyse the putative alterations in three-dimensional mPTP structures involved in MeHg poisoning and their relationship with mPTP and F-ATPase activities.

We did not find literature reports of MeHg concentrations in human hearts related to the cardiotoxicity findings shown in this work. In addition to species-specific differences, the mice in this study did not receive MeHg via food for a long time or throughout their lifetime, unlike riverside residents and gold mine workers. Despite this limitation, our study calls attention to the importance of performing periodic cardiovascular examinations in MeHg-exposed populations.

## 4. Conclusions

**Figure 8:**
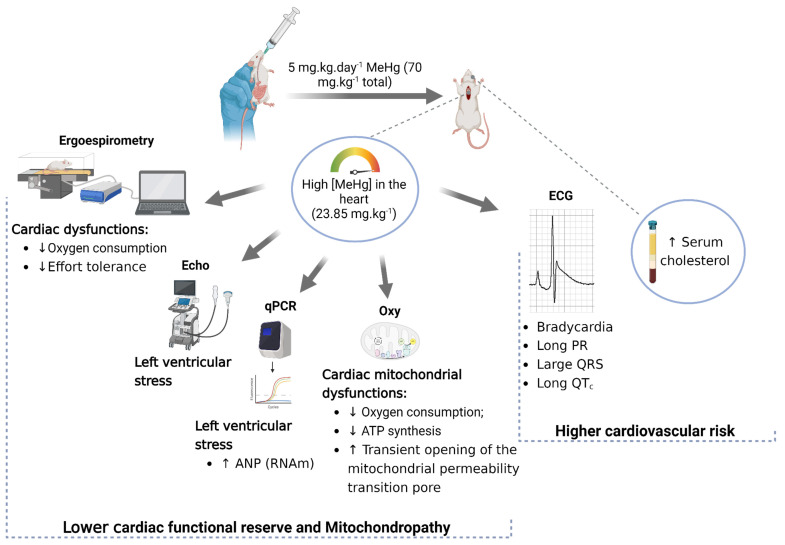
Summary of cardiac dysfunctions caused by methylmercury intoxication.

The patterns of ex vivo heart mitochondrial dysfunction, such as a lower respiratory control ratio and ATP production, presented by mice poisoned with a subacute dose of MeHg were correlated with heart derangement in vivo in two ways: lower peak oxygen consumption during running tests and impaired left ventricle function assessments by echocardiography. These results, displayed in Figure 8, revealed a worrisome cardiovascular death risk factor for MeHg exposure, as witnessed in Amazon riverine populations, which eat large amounts of MeHg-contaminated fishes. Additionally, mercury is one of the top ten chemical concerns for public health, and the model described herein can serve as an instrument for the in vivo testing of strategies, pharmacological or not, for cardioprotection.

## Figures and Tables

**Table 1:** Systemic MeHg toxicity parameters and MeHg concentration in heart samples.

	Pretest	Control	MeHg70
Body weight (g) (N = 25)	18.31 ± 0.28	20.41 ± 0.34	17.48 ± 0.65 *
Survival rate (%) (N = 25)	100	95.23	46.40
[MeHg] in heart tissue samples (N = 5)	unchecked	0.01	23.85 ± 2.62 ***

MeHg concentration data are expressed as mg.kg^−1^. * *p* < 0.05 and *** *p* < 0.001 when comparing the MeHg70 group with the control group. Data are mean ± SE.

**Table 2:** Electrocardiography measurements.

	Pretest (Awake) N = 33	Anaesthetised		Awake	
		Control N = 17	MeHg70 N = 11	Control N = 11	MeHg70 N = 20
Heart rate (BPM)	614 ± 13	208 ± 13	237 ± 8	634 ± 20	522 ± 24 **
P wave duration (ms)	11.09 ± 0.68	11.68 ± 1.05	10.75 ± 1.42	10.43± 0.67	10.37 ± 0.66
PR interval (ms)	27.80 ± 0.45	34.76 ± 0.49	44.27 ± 0.48 **	28.73 ± 0.73	28.33 ± 1.09
QRS complex duration (ms)	9.02 ± 0.28	9.92 ± 0.33	10.11 ± 0.34	9.19 ± 0.33	10.27± 0.25 *
QTc (ms)	71.02 ± 1.55	50.51 ± 0.41	79.36 ± 0.75 **	68.96 ± 3.41	88.91 ± 3.32 ***

* *p* < 0.05; ** *p* < 0.01; *** *p* < 0.001; when data from MeHg70 groups were compared to counterpart control groups. Data are mean ± SE. BPM = beats per min.

**Table 3:** Echocardiography measurements.

	Pretest	Control	MeHg70
Heart rate (BPM)	133 ± 13	130 ± 10	135 ± 18
LVAWDT (mm)	0.50 ± 0.0	0.48 ± 0.0	0.40 ± 0.0 **
LVDD (mm)	3.61 ± 0.01	3.63 ± 0.00	3.42 ± 0.01
LVSD (mm)	1.31 ± 0.0	1.37 ± 0.0	1.70 ± 0.0 *
SV (mL)	0.05 ± 0.0	0.05 ± 0.0	0.04 ± 0.0
Ao/LA	0.92 ± 0.01	0.97 ± 0.01	0.85 ± 0.03 **
EF (%)	94.50 ± 0.8	94.17 ± 0.7	86.14 ± 1.90 ***
CO (mL/min)	7.37 ± 0.75	6.39 ± 1.00	5.05 ± 1.04
IVRT (ms)	unchecked	36.83 ± 2.99	36.00± 6.10
E/A	4.42 ± 0.49	3.87 ± 0.48	4.84 ± 1.38

Data are mean ± SE. BPM = beats per minute; LVAWDT = left ventricular anterior wall diastolic thickness; LVDD = left ventricle diastolic diameter; LVSD = left ventricular systolic diameter; SV = stroke volume; Ao/LA = aorta/ left atrium ratio; EF = ejection fraction; CO = cardiac output; IVRT = isovolumic relaxation time; E/A = E wave/A wave ratio. (N = 8). for * *p* < 0.05; ** *p* < 0.01; *** *p* < 0.001 when data from animals from the MeHg group were compared to animals from the control group.

**Table 4:** Enzymatic activity assays in heart tissue homogenates.

	Control Group	MeHg70
Citrate synthase (CS, U.mg^−1^.min^−1^), N= 4	1042 ± 332.1	1106 ± 97.9
Succinate dehydrogenase (SD, [pmol.mg^−1^.s^−1^]/CS/g wet tissue), N = 6	14.53 ± 4.08	14.83 ± 5.42
ATPase activity of complex V responsive to azide (ATPase, U.mg^−1^/CS), N = 4	4.45 ± 0.8	3.21 ± 0.7

Data represents mean ± SE.

## Data Availability

The data presented in this study are available on request from the corresponding author.
